# Toward a Novel Phenotypic Classification of Parkinson's Disease for Rehabilitation: A Case Series

**DOI:** 10.7759/cureus.112482

**Published:** 2026-07-11

**Authors:** Junya Ogawa, Yuya Yamaguchi, Kei Kakegawa, Tadamitsu Matsuda

**Affiliations:** 1 Parkinson's Disease Rehabilitation, PDit Studio Ginza, Smile Space Inc., Tokyo, JPN; 2 Neurorehabilitation, Health and Neuro Training ASPATH, Kagoshima, JPN; 3 Graduate School of Human Life Design, Toyo University, Tokyo, JPN; 4 Graduate School of Health Science, Juntendo University, Tokyo, JPN

**Keywords:** early intensive training, movement-based typology, parkinson's disease, rehabilitation, subtypes

## Abstract

Traditional classifications of Parkinson's disease (PD), such as tremor-dominant and postural instability/gait difficulty (PIGD) types, are useful for prognosis but offer limited guidance for individualized interventions. This case series descriptively explores clinically observed movement patterns, referred to as rigid, loose, and hemi types, to illustrate how rehabilitation strategies were adapted for each case.

Three individuals with PD were categorized by their predominant motor presentation and completed a three-month intensive rehabilitation program. The rigid type received gait training with visual feedback and rhythmic cueing. The loose type underwent trunk- and hip-focused strengthening combined with aerobic training to improve movement control. The hemi type received laterality-focused sensorimotor training progressing from single-joint to coordinated multi-joint movements.

All participants showed improvements in gait symmetry, postural control, and motor function, with reductions in the Movement Disorder Society-Unified Parkinson's Disease Rating Scale (MDS-UPDRS) Part III and Parkinson's Disease Questionnaire-39 (PDQ-39) scores and enhanced spatiotemporal gait parameters.

A movement-based perspective may serve as a practical clinical framework for considering individualized rehabilitation strategies. These findings should be interpreted as exploratory and hypothesis-generating.

## Introduction

Globally, the number of people with Parkinson's (PwP) is expected to increase considerably, reaching 13.6 million by 2040 [1]. Although Parkinson's disease (PD) is currently classified as a rare disease, its increasing prevalence suggests that it may soon affect a much broader population. PD is a neurodegenerative disorder primarily characterized by motor symptoms, including bradykinesia, resting tremor, muscle rigidity, and postural reflex impairment. In addition to these motor impairments, non-motor symptoms such as olfactory dysfunction, constipation, and depression are frequently observed. As symptoms progress, they lead to a decline in activities of daily living (ADL) and overall quality of life [2]. Even in the early stages of PD, within five years of diagnosis, many individuals seek to achieve improvements in gait, balance, and resting tremors. Recognizing the early emergence of symptoms is essential for guiding treatment strategies [3]. Current treatments for PD, which include pharmacological, non-pharmacological, and surgical interventions, primarily focus on symptom management. Pharmacological treatments commonly involve dopamine-related medications, such as carbidopa-levodopa and dopamine agonists. Surgical interventions included carbidopa-levodopa intestinal infusion therapy and deep brain stimulation (DBS). Non-pharmacological approaches include rehabilitation therapies, such as physical, occupational, and speech therapies, as well as structured exercise programs [4].

The motor symptoms of PD vary widely among individuals, and several subtyping approaches have been proposed. Many previous studies have classified patients using the Unified Parkinson's Disease Rating Scale (UPDRS)-based assessments or genetic analyses [5]; however, these symptom-oriented methods do not adequately capture qualitative movement characteristics relevant to rehabilitation [6]. Classical clinical subtypes [7], including tremor-dominant (TD), postural instability/gait difficulty (PIGD), and indeterminate types, typically derived from UPDRS or Movement Disorder Society (MDS)-UPDRS motor item scores, likewise provide limited insight into movement strategies during functional tasks. The TD subtype is characterized by predominant resting tremor with relatively preserved axial motor function, whereas the PIGD subtype is defined by greater impairments in gait, balance, and postural control and is associated with higher risks of falls and functional decline, yet primarily reflects symptom severity rather than task-specific movement strategies. More recently, subgroups based on quantitative gait features have been explored, but these data-driven approaches have yet to demonstrate clear applicability in guiding individualized, movement-based interventions. Several studies have previously investigated the effects of physical therapy, a key component of rehabilitation, on PWP [8,9]. One meta-analysis examining the impact of physical therapy on physical function, psychological well-being, and quality of life in patients with PWP demonstrated considerable improvements in mobility, health-related quality of life, and walking ability [10]. Another meta-analysis focused on the effects of long-term physical therapy (≥6 months) in patients with early- to mid-stage PD. The results showed notable improvements in motor symptoms during the "off" phase and a reduction in antiparkinsonian medication dosage, suggesting that long-term physical therapy may help manage symptoms and reduce the risks associated with pharmacological treatment. These findings highlight the importance of incorporating physical and exercise therapies into pharmacological treatments to slow disease progression in PwP.

However, selecting an appropriate rehabilitation strategy requires more than an assessment of symptom severity alone. Physical therapists must also evaluate how movement impairments are expressed during functional activities, as movement quality directly influences the choice of therapeutic exercises and motor learning strategies.

In this study, we explored three movement-based phenotypic patterns, rigid, loose, and hemi, representing distinct movement characteristics observed during functional activities, as an exploratory framework for characterizing movement quality in PD.

These author-proposed movement-based phenotypic patterns are preliminary and require further validation before they can be considered an established phenotypic classification.

Therefore, the purpose of this study was to explore an exploratory movement-based phenotypic classification by identifying characteristic movement patterns during functional tasks (gait and sit-to-stand) in individuals with early-stage PD.

## Case presentation

Participants

Three individuals with mild PD participated in this study. All participants were in their 50s and had been diagnosed with PD between 2017 and 2020. Hoehn and Yahr (HY) stages ranged from 1 to 2. None had major comorbid neurological or orthopedic conditions that would interfere with exercise participation. Baseline motor symptoms included bradykinesia, rigidity, gait disturbances, and laterality, although the specific presentation varied among cases. Details of the individual characteristics and presenting symptoms for each participant are presented in the case descriptions. All evaluations were performed while the participants were in the ON medication state.

Measurements

To evaluate the changes associated with the three-month intervention, four outcome measures were obtained before and after training: (1) MDS-UPDRS Part III, a clinician-rated measure assessing motor symptom severity; (2) Timed Up and Go (TUG), a functional mobility test that reflects dynamic balance, gait initiation, and turning ability; (3) step length (cm), calculated from inertial measurement unit (IMU)-based gait analysis during a five-minute walking task; and (4) Parkinson's Disease Questionnaire-39 (PDQ-39), a patient-reported measure of health-related quality of life. The intervention included twice-weekly in-person personalized training with a physical therapist and additional home-based self-training with video-based feedback.

Established minimal clinically important differences (MCIDs) were used as reference values for the descriptive interpretation of the magnitude of change. For the TUG test, the minimal detectable change (MDC) is 3.5 seconds [11], indicating the threshold beyond which a change is unlikely to reflect measurement error. For motor severity, an improvement of ≥3.25 points on the MDS-UPDRS Part III has been reported as the MCID [12]. For gait amplitude, an increase in step length of at least 3.6 cm represents a clinically meaningful change [13]. For quality of life, a reduction of ≥12.7 points on the PDQ-39 summary index is considered clinically meaningful [14]. These MDC and MCID values were derived from previous studies using anchor-based or distribution-based approaches. While some estimates were based on patient-reported outcomes (e.g., PDQ-39), others, such as those for MDS-UPDRS Part III, were anchored to clinician-rated scales, reflecting more objective assessments of change. As these values may vary depending on disease stage, anchor selection, and evaluation context, they should be interpreted with caution.

Movement-Based Type Description

Unlike conventional PD subtypes based on symptom severity or UPDRS scores, the movement types described here are based on clinically observed movement characteristics during functional tasks. Participants were classified into three types based on the predominant movement characteristics observed during gait assessment: rigid type, reduced arm swing, decreased trunk and shoulder rotation, and short stride length; loose type, relatively preserved stride length accompanied by increased postural sway, instability, and variable movement timing; and hemi type, marked laterality in step length, arm swing, trunk lean, or lower-limb recruitment. Type assignment was performed by experienced physical therapists through comprehensive clinical evaluation, integrating movement observation, neurological findings, and functional performance. Because this was an exploratory case series, no standardized scoring system or quantitative classification threshold was applied. The proposed framework represents an initial step toward developing an objective movement-based phenotypic classification and requires further validation using standardized assessment methods.

The classification was primarily based on clinical observation, while quantitative IMU data were reviewed as supplementary information to confirm the observed movement characteristics. As quantitative IMU data are not presented in this report, the classification should be interpreted based on the clinical characteristics described above. As this is a preliminary study, these definitions should be regarded as an exploratory framework requiring further refinement and validation.

Case presentation

Case 1 (Rigid Type)

We present the case of a woman in her 50s. Her initial symptoms were dragging her feet when walking, and she was diagnosed with PD seven years before the intervention. The main symptoms were pronounced bradykinesia, increased movement difficulty during dual-task activities, and trunk-centered muscle rigidity. She worked as a writer and independently traveled by train with her husband to a studio where she exercises. Her main complaints included difficulty lifting her feet, frequent ankle catching, slower walking speed, and difficulty performing smooth name card exchanges.

**Figure 1:**
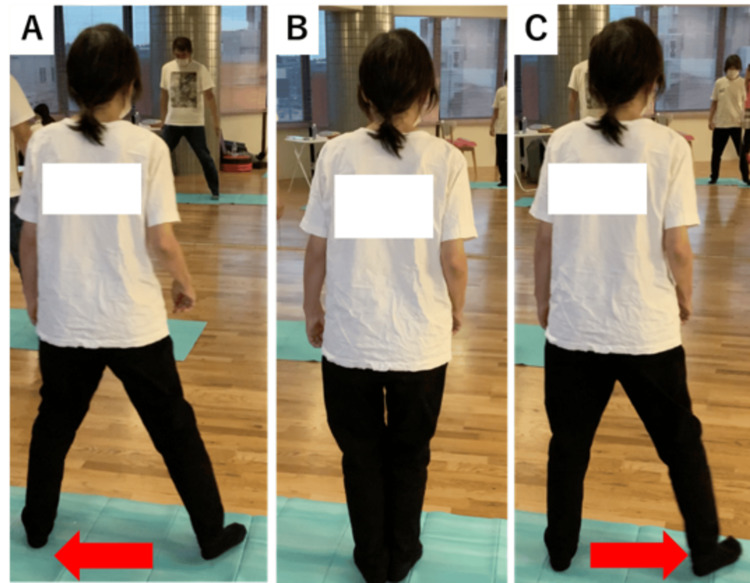
Repeated side-step training with rhythmic cueing and visual feedback (A) Left side-step, (B) neutral standing position, and (C) right side-step during repeated side-step training. The exercise was performed using a metronome to provide external rhythmic cues, beginning at approximately 80 bpm and progressing to approximately 100 bpm as the participant became accustomed to the task. Throughout the exercise, the participant was instructed to maintain full movement amplitude. Movement patterns were recorded using a tablet device and reviewed to provide visual feedback on movement amplitude. Corrective instructions were given when the amplitude was narrower than the target range.

Based on the predominant movement characteristics observed during gait assessment, this participant was classified as the rigid type. According to the MDS-UPDRS tremor/PIGD ratio method [15], she was classified as TD. Therefore, rehabilitation focused on improving movement amplitude, trunk rotation, and rhythmic gait. The intervention program is summarized in Table 1, and an example of the aerobic stepping exercise is shown in Figure 1.

**Table 1:** Exercise intervention for Case 1

Contents	Methods and effects
Rhythmic aerobic exercise (to prioritize large movement execution) (Figure 1)	Enhances muscle relaxation and improves ease of movement; includes exaggerated step walking and stepping exercises
Dual-task training	Aims to improve movement efficiency under cognitive load, for example, walking while performing hand tasks (e.g., simulated name card exchange movements)
Proprioceptive and sensory-motor training	Includes ankle dorsiflexion exercises and weight-shifting drills to address ankle catching issues
Gait training with visual and rhythmic feedback	Utilizes a tablet to provide video feedback on walking patterns. A metronome (starting at 80 bpm and gradually increasing to 120 bpm) was used to enhance gait rhythm and speed control

Outcome: Following the three-month intervention, improvements were observed in foot clearance, movement fluidity, and step length. The step length increased from 47.6 cm to 55.6 cm, whereas gait speed remained largely unchanged (1.14-1.12 m/s). The participant reported the following: "I can now take larger steps when walking" and "Standing up from a chair and walking at restaurants has become easier".

Case 2 (Loose Type)

We present the case of a man in his 50s. His initial symptom was micrographia, and he was diagnosed with PD four years before the intervention. His HY classification was 2. His main symptoms were trunk swaying during walking and an inconsistent step width for each stride. Additionally, his walking and movement paces were excessively fast. He worked as a company employee and attended the studio on his own during training. His main complaints were as follows: "When carrying shopping bags or holding items with both hands, my unsteadiness worsens", "Bowling is my hobby, but I feel like my stamina has decreased-I get tired after just two games", and "I frequently experience fatigue and instability while standing and walking". The results of the manual muscle strength tests for the lower limbs and trunk were as follows (right/left): hip extension, 3/3; hip abduction, 3/3; scapular retraction, 3/4; and trunk flexion, 4.

The patient appeared to have reduced awareness of deep muscle contractions, particularly in the deep abdominal, gluteus maximus, and rhomboid muscles, based on clinical observation. When comparing the perceived contraction intensity of the rectus abdominis (superficial muscle) to 10, the deep muscles were rated 5. He further exhibited difficulty in selectively contracting the deep trunk muscles. When attempting postural control exercises requiring abdominal muscle contractions, such as bilateral leg raises in the supine position, the patient struggled to maintain proper lumbar positioning and was unable to sustain a neutral lumbar curve.

The patient also presented with weakness and sensory deficits in the gluteal muscles, which contributed to excessive movement speed and reduced movement quality. During hip extension, he tended to compensate with excessive lumbar extension and the overactivation of the lumbar extensors.

Based on the predominant movement characteristics observed during gait assessment, this participant was classified as the loose type. According to the MDS-UPDRS tremor/PIGD ratio method [15], he was classified as PIGD. These findings suggested impaired trunk stability and reduced sensory awareness rather than insufficient movement amplitude. Therefore, rehabilitation focused on improving trunk stability, gluteal muscle activation, sensory awareness, and gait control. The intervention program is summarized in Table 2.

**Table 2:** Exercise intervention for Case 2

Contents	Methods and effects
Enhancement of sensory input	Facilitating muscle contraction awareness in the gluteal and deep trunk muscles using manual resistance; introducing proprioceptive neuromuscular facilitation patterns to improve pelvic stability
Trunk stabilization training	Activating deep abdominal muscles through draw-in exercises; performing supine leg-raising exercises to reinforce appropriate trunk stabilization
Gluteal muscle strengthening	Bridge exercises with attention to proper pelvic alignment; side planks to enhance coordination between the gluteal and trunk muscles
Gait pattern improvement	Gait speed control training utilizing visual and auditory feedback; balance exercises incorporating real-life scenarios, such as carrying objects while walking

Outcome: Following the intervention, improvements were observed in walking stability and endurance. The MDS-UPDRS Part III score improved from 24 to 6, while the PDQ-39 score improved from 61 to 53. The participant reported the following: "My walking stability has improved" and "I feel less fatigued even after prolonged movement, making it easier to play my favorite sport, bowling".

Case 3 (Hemi Type)

We present the case of a woman in her 50s. Her initial symptom was a sensation of weakness in the left lower limb during gait, and she was diagnosed with PD three years before the intervention. Her HY classification was 1. The main symptoms were pronounced muscle weakness, bradykinesia, rigidity, and restricted joint range of motion in the left upper and lower limbs compared to the right limbs. She worked full-time in clerical duties. She commuted independently to the rehabilitation studio using public transportation. Her main complaints were as follows: "I tend to drag my left foot while walking" and "My left leg feels stiff and tight".

Clinical findings indicated that symptoms were present in the left upper limb, lower limb, and trunk. The MDS-UPDRS Part III score, covering tremor, rigidity, and bradykinesia items, was 2 points on the right side and 13 points on the left side. Gait assessment revealed reduced backward swing of the left upper limb, limitations in left hip flexion and extension, and decreased ankle dorsiflexion and plantarflexion on the left side. Sensory evaluation showed markedly reduced awareness of left abdominal muscle contraction. Although muscle contraction was confirmed by manual palpation during specific tasks, the subjective sensation was rated as 2/10 on the left side and 10/10 on the right side. Manual muscle testing demonstrated left-sided weakness (right/left): hip flexion, 5/2; hip extension, 4/2; ankle plantarflexion, 4/3; and ankle dorsiflexion, 4/2.

**Figure 2:**
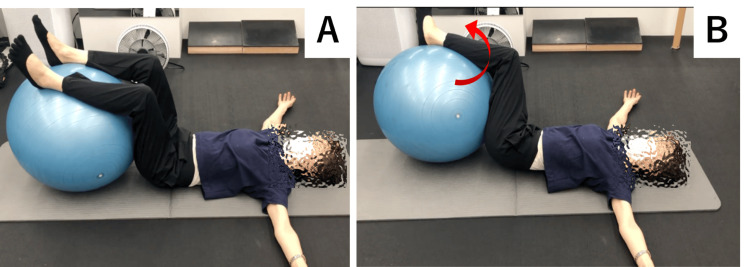
Supine abdominal training using a balance ball (A) Starting position, with both lower limbs placed on a balance ball in the supine position. (B) The lower trunk and lower limbs were tilted to the right to stretch the left abdominal region, followed by the activation of the left abdominal muscles to return to the starting position. This exercise was used to facilitate awareness and the activation of the left abdominal muscles. As the participant became accustomed to abdominal muscle contraction in the supine position, the difficulty was progressively increased by incorporating kneeling and standing exercises with upper trunk rotation.

**Figure 3:**
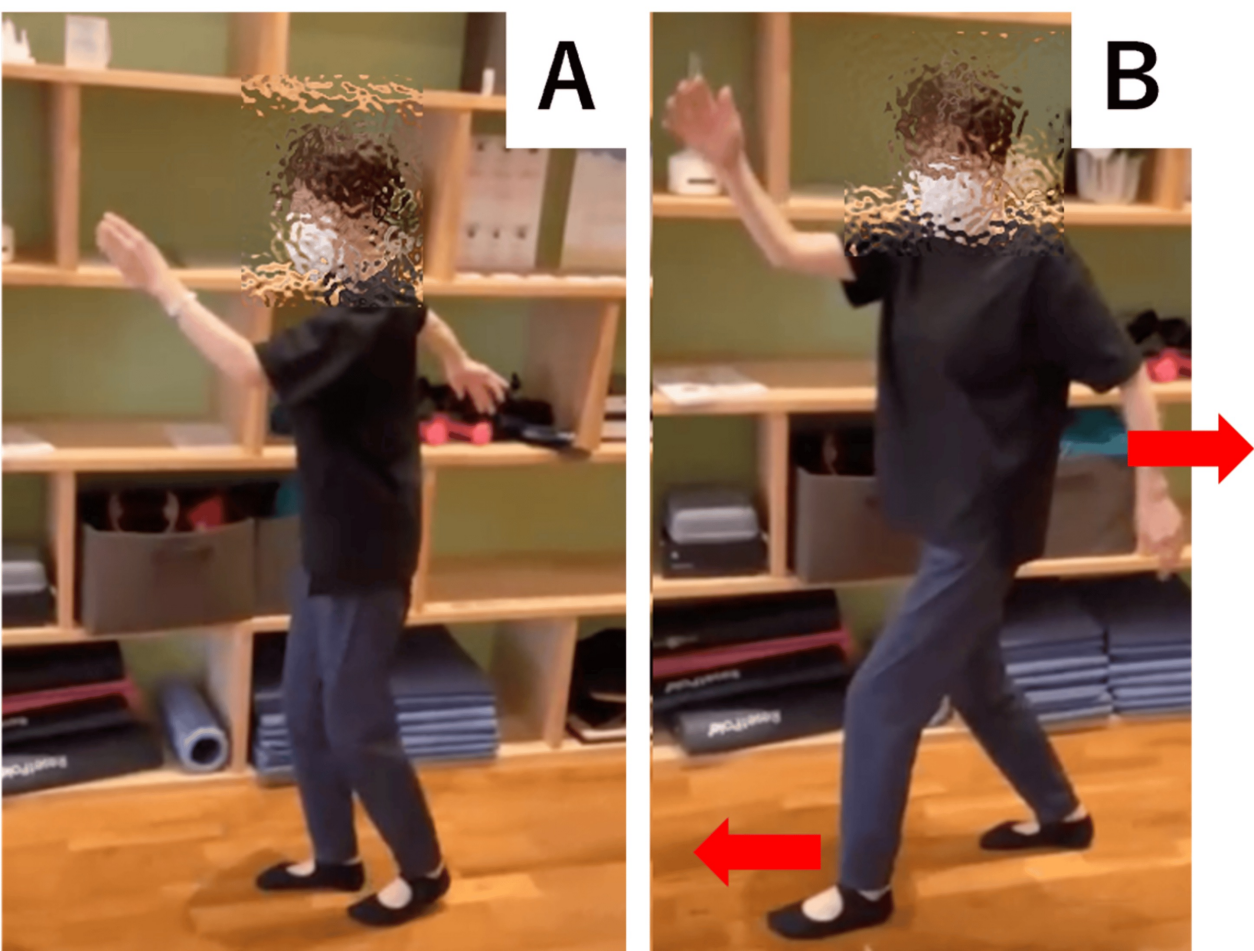
Arm swing and gait-related coordination training (A) Initial stepping posture before left shoulder extension. (B) Stepping with simultaneous left shoulder extension to promote scapular retraction and trunk rotation. This exercise was used to improve coordinated arm swing and gait-related trunk movement on the affected side. Training progressed from isolated shoulder extension to more complex task-oriented practice incorporating scapular retraction, trunk rotation, and step coordination.

Based on the predominant movement characteristics observed during gait assessment, this participant was classified as the hemi type. According to the MDS-UPDRS tremor/PIGD ratio method [15], she was classified as indeterminate. These findings indicated marked laterality in motor performance and sensory awareness. Therefore, rehabilitation focused on improving left-sided trunk stability, sensory awareness, arm swing, and gait coordination. The intervention program is summarized in Table 3, and representative exercises are shown in Figure 2 and Figure 3.

**Table 3:** Exercise intervention for Case 3

Contents	Methods and effects
Abdominal muscle training (Figure 2)	Progressive exercises were implemented, with a particular focus on the left abdominal muscles
Gluteal muscle training	Progressive gluteal muscle training was introduced, with a focus on the left side
Arm swing and gait training (Figure 3)	Progressive training focused on improving the left-side arm swing during gait
Sensory-motor integration emphasis	Throughout training, emphasis was placed on sensory feedback, rather than repetitive movements. Participants were asked to answer the following questions: "Do you feel activation in the left side of your abdomen?" and "Can you sense your thigh moving backward from the hip joint?". This approach aims to enhance both motor and sensory pathways and facilitate motor learning through improved proprioceptive feedback

Outcome: Following the intervention, improvements were observed in left hip flexion and extension range of motion and left arm swing during gait. The left-sided MDS-UPDRS Part III score improved from 13 to 6. The participant commented the following: "I no longer drag my left foot when walking" and "My left arm now swings naturally and more prominently during walking".

Table 4 shows the changes in each subject before and after the intervention. All participants completed a three-month intervention period. Supervised training sessions were conducted twice per week, and all participants additionally performed home-based exercises at least twice per week.

**Table 4:** Clinical outcomes of the three movement-based types following individualized rehabilitation Pre→post; * indicates a change exceeding the previously reported MCID or MDC described under "Measurements". MDS-UPDRS: Movement Disorder Society-Unified Parkinson's Disease Rating Scale; TUG: Timed Up and Go; PDQ-39: Parkinson's Disease Questionnaire-39

	Rigid type (Case 1)	Loose type (Case 2)	Hemi type (Case 3)
MDS-UPDRS Part III (score)	19→17	24→6*	18→10*; right 2→2, left 13→6
Gait speed (m/sec)	1.14→1.12	1.54→1.93	1.30→1.32
Step length (cm)	47.6→55.6*	62.5→66.7*	62.5→62.5
TUG (sec)	7.0→8.2	6.7→5.4	7.1→7.8
PDQ-39 (score)	75→74	61→53	108→79*

## Discussion

Across the three cases in this study, changes were observed in multiple outcome measures; however, whether these changes exceeded the MCID and MDC varied depending on the measure. Improvements in MDS-UPDRS Part III and step length exceeded the MCID in two cases, suggesting that these changes may be clinically meaningful. It should be noted that the MCID for MDS-UPDRS Part III is typically derived using clinician-rated anchors, reflecting more objective assessments of change. In contrast, although PDQ-39 scores also changed, only Case 3 exceeded the MCID threshold when considering previously reported values for the PDQ-39 total score. This MCID was derived using anchor-based approaches based on patient-reported outcomes and therefore may be influenced by patient perception and contextual factors in daily life. In addition, the study population from which this MCID was derived included patients with varying and relatively advanced disease severity, which may differ from the characteristics of the present cases. Accordingly, the interpretation of clinically meaningful change in PDQ-39 should be made with caution. Furthermore, changes in the TUG test did not exceed the established MDC in any of the cases. However, this finding should be interpreted with caution. In PD, a longer TUG time does not necessarily indicate poorer motor performance, as improvements in movement quality, including enhanced postural control, safer turning strategies, or smoother movement execution, may require slower but more controlled movements. Therefore, quantitative outcome measures alone may not fully capture clinically meaningful changes, highlighting the importance of incorporating qualitative movement assessment into rehabilitation evaluation.

Previous studies [8-10] have demonstrated that early and intensive physiotherapy can improve UPDRS and TUG scores and that exercise has beneficial effects on physical function, quality of life, muscle strength, balance, and gait performance in individuals with PD. In the present study, similar trends of improvement were observed across these outcomes, which are generally consistent with previous findings. However, unlike conventional approaches that primarily focus on symptom severity, the present study individualized interventions based on movement type. This approach enabled the application of targeted strategies tailored to the specific movement characteristics of each case, representing a key distinction from standard physiotherapy. However, given the marked heterogeneity of motor symptoms in PD, the rationale for adopting a movement-type-based approach warrants further consideration.

PD exhibits marked heterogeneity in motor symptoms [3], and several subtype classifications, such as TD and PIGD, have been proposed based on UPDRS scores. These classifications are useful for prognosis but provide limited guidance for selecting movement-based rehabilitation strategies.

In rehabilitation settings, symptom-based subtypes do not always translate into practical guidance for exercise prescription. While physicians prioritize quantitative symptom assessment for diagnosis, rehabilitation professionals focus on qualitative movement characteristics to support functional improvement. From this perspective, organizing patients according to predominant movement patterns may facilitate individualized rehabilitation planning.

Previous studies using quantitative gait analysis have demonstrated the existence of diverse walking patterns in PD, independent of disease severity.

The specific features of each movement type are detailed below.

**Figure 4:**
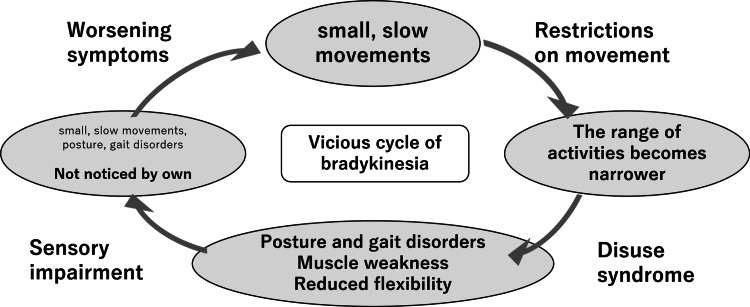
The negative cycle of bradykinesia This figure illustrates a conceptual cycle in which reduced movement amplitude and awareness of movement changes may contribute to worsening bradykinesia and functional decline. This illustration was created by the authors using Microsoft PowerPoint (Microsoft Corp., Redmond, WA, USA).

The rigid type, as exemplified by Case 1 in this study, was characterized by shortened stride length and restricted trunk and shoulder rotation. Although gait speed showed minimal change in the rigid type, improvements were observed in movement fluidity and step length, suggesting qualitative rather than purely quantitative changes. Reduced awareness of gradual movement changes may contribute to a cycle of bradykinesia and functional decline, as conceptually illustrated in Figure 4. The conceptual model shown in Figure 4 is based on the present cases and previous literature and is intended to illustrate a potential clinical mechanism rather than an experimentally validated pathway. Future studies are needed to verify this proposed mechanism using objective movement analysis and longitudinal observations.

The loose type, as exemplified by Case 2, was characterized by relatively preserved stride length accompanied by subjective instability and difficulty regulating movement speed. Based on clinical observation, reduced awareness of deep muscle activation and impaired rhythm control may contribute to movement variability. These features partially overlap with the characteristics of the PIGD subtype.

The hemi type, as exemplified by Case 3, was characterized by persistent lateralized motor and sensory impairments. Reduced awareness of asymmetric movement patterns may contribute to gait asymmetry and functional limitations. In Case 3, interventions targeting the affected side were associated with reduced asymmetry.

The rehabilitation programs in this study incorporated evidence-based strategies, including trunk-focused training, progressive task difficulty, and short-term intensive practice [16-18]. Such approaches have been reported to improve balance, gait, and motor learning in individuals with PD. Tailoring these strategies according to predominant movement characteristics may enhance their clinical effectiveness [19,20].

Importantly, this intervention was tailored based on the patient's movement type. Given the heterogeneity of Parkinsonian symptoms, movement-based classification enables more individualized and targeted rehabilitation strategies [3], which may lead to better outcomes and improved daily functioning.

Classifying PwPs into different movement-based types can facilitate the selection and implementation of appropriate training and exercise therapies. This section proposes suitable training methods for PwP of the rigid, loose, and hemi types (Table 5).

**Table 5:** Examples of training approaches for each type

Movement-based types	Recommended training approaches
Rigid type	Use of tablet-based video feedback to raise gait awareness; walking programs with step-count goals; progressive gait pacing using a metronome (80-140 bpm); repetitive gait training with external cues; torso rotation stretches to improve the flexibility of oblique and pectoral muscles
Loose type	Core and hip muscle strengthening (e.g., transverse abdominis, internal oblique, gluteus maximus/medius); progressive resistance training with bands (≥3 seconds per rep); metronome pacing at 60-80 bpm to reduce excessive speed; incorporate moderate- to high-intensity aerobic activities such as step-ups and large-stride walking
Hemi type	Begin with single-joint activation on the symptomatic side; transition to multi-joint exercises as symmetry improves; increase training load and complexity gradually according to the principle of progressive overload

The rigid, loose, and hemi movement-based phenotypic patterns proposed in this study represent an exploratory framework derived from clinical observation. Although they were developed to facilitate rehabilitation planning, they are not intended to replace established PD subtypes at this stage.

Future studies involving larger cohorts are needed to establish standardized classification criteria and evaluate reproducibility and inter-rater reliability. In addition, integrating quantitative movement analysis using wearable or markerless sensing technologies with standardized clinical checklists and AI-assisted classification methods may provide a more objective and reproducible approach to movement-based phenotypic classification. Such developments will help determine the clinical validity and applicability of the proposed framework.

In addition, adherence to the home exercise program was not objectively monitored; therefore, its contribution to the observed outcomes could not be determined. Potential confounding factors, such as medication status and daily physical activity, were also not evaluated in the present study. These factors may have influenced the observed outcomes and should be incorporated into future studies to strengthen the interpretation and validation of the proposed movement-based phenotypic framework.

## Conclusions

This case series descriptively explored three movement-based types of PD, rigid, loose, and hemi, and illustrated how rehabilitation strategies were adapted according to their predominant movement characteristics. Although preliminary, this movement-based framework may provide a practical perspective for individualized rehabilitation planning beyond conventional symptom-based classifications. Further studies with larger samples and quantitative validation are needed to examine the reliability, clinical utility, and generalizability of this proposed classification.
